# Tumor-associated copy number changes in the circulation of patients with prostate cancer identified through whole-genome sequencing

**DOI:** 10.1186/gm434

**Published:** 2013-04-05

**Authors:** Ellen Heitzer, Peter Ulz, Jelena Belic, Stefan Gutschi, Franz Quehenberger, Katja Fischereder, Theresa Benezeder, Martina Auer, Carina Pischler, Sebastian Mannweiler, Martin Pichler, Florian Eisner, Martin Haeusler, Sabine Riethdorf, Klaus Pantel, Hellmut Samonigg, Gerald Hoefler, Herbert Augustin, Jochen B Geigl, Michael R Speicher

**Affiliations:** 1 Institute of Human Genetics, Medical University of Graz, Harrachgasse 21/8, A-8010 Graz, Austria; 2 Department of Urology, Medical University of Graz, Auenbruggerplatz 5/6, A-8036 Graz, Austria; 3 Institute for Medical Informatics, Statistics and Documentation, Medical University of Graz, Auenbruggerplatz 2, A-8036 Graz, Austria; 4 Institute of Pathology, Medical University of Graz, Auenbruggerplatz 25, A-8036 Graz, Austria; 5 Division of Oncology, Medical University of Graz, Auenbruggerplatz 15, A-8036 Graz, Austria; 6 Department of Obstetrics and Gynecology, Medical University of Graz, Auenbruggerplatz 14, A-8036 Graz, Austria; 7 Institute of Tumor Biology, University Medical Center Hamburg Eppendorf, Martinistr. 52, D-20246 Hamburg, Germany

## Abstract

**Background:**

Patients with prostate cancer may present with metastatic or recurrent disease despite initial curative treatment. The propensity of metastatic prostate cancer to spread to the bone has limited repeated sampling of tumor deposits. Hence, considerably less is understood about this lethal metastatic disease, as it is not commonly studied. Here we explored whole-genome sequencing of plasma DNA to scan the tumor genomes of these patients non-invasively.

**Methods:**

We wanted to make whole-genome analysis from plasma DNA amenable to clinical routine applications and developed an approach based on a benchtop high-throughput platform, that is, Illuminas MiSeq instrument. We performed whole-genome sequencing from plasma at a shallow sequencing depth to establish a genome-wide copy number profile of the tumor at low costs within 2 days. In parallel, we sequenced a panel of 55 high-interest genes and 38 introns with frequent fusion breakpoints such as the *TMPRSS2-ERG *fusion with high coverage. After intensive testing of our approach with samples from 25 individuals without cancer we analyzed 13 plasma samples derived from five patients with castration resistant (CRPC) and four patients with castration sensitive prostate cancer (CSPC).

**Results:**

The genome-wide profiling in the plasma of our patients revealed multiple copy number aberrations including those previously reported in prostate tumors, such as losses in 8p and gains in 8q. High-level copy number gains in the *AR *locus were observed in patients with CRPC but not with CSPC disease. We identified the *TMPRSS2-ERG *rearrangement associated 3-Mbp deletion on chromosome 21 and found corresponding fusion plasma fragments in these cases. In an index case multiregional sequencing of the primary tumor identified different copy number changes in each sector, suggesting multifocal disease. Our plasma analyses of this index case, performed 13 years after resection of the primary tumor, revealed novel chromosomal rearrangements, which were stable in serial plasma analyses over a 9-month period, which is consistent with the presence of one metastatic clone.

**Conclusions:**

The genomic landscape of prostate cancer can be established by non-invasive means from plasma DNA. Our approach provides specific genomic signatures within 2 days which may therefore serve as 'liquid biopsy'.

## Background

Prostate cancer is the most common malignancy in men. In Europe each year an estimated number of 2.6 million new cases is diagnosed [1]. The wide application of PSA testing has resulted in a shift towards diagnosis at an early stage so that many patients do not need treatment or are cured by radical surgery [2]. However, patients still present with metastatic or recurrent disease despite initial curative treatment [3]. In these cases prostate-cancer progression can be inhibited by androgen-deprivation therapy (ADT) for up to several years. However, disease progression is invariably observed with tumor cells resuming proliferation despite continued treatment (termed castration-resistant prostate cancer or CRPC) [4]. CRPC is a strikingly heterogeneous disease and the overall survival can be extremely variable [5]. Scarcity of predictive and prognostic markers underlines the growing need for a better understanding of the molecular makeup of these lethal tumors.

However, acquiring tumor tissue from patients with metastatic prostate cancer often represents a challenge. Due to the propensity of metastatic prostate cancer to spread to bone biopsies can be technically challenging and limit repeated sampling of tumor deposits. As a consequence, considerably less is understood about the later acquired genetic alterations that emerge in the context of the selection pressure of an androgen-deprived milieu [6].

Consistent and frequent findings from recent genomic profiling studies in clinical metastatic prostate tumors include the *TMPRSS2-ERG *fusion in approximately 50%, 8p loss in approximately 30% to 50%, 8q gain in approximately 20% to 40% of cases, and the androgen receptor (*AR*) amplification in approximately 33% of CRPC cases [7-10]. Several whole-exome or whole-genome sequencing studies consistently reported low overall mutation rates even in heavily treated CRPCs [9-14].

The difficulties in acquiring tumor tissue can partly be addressed by elaborate procedures such as rapid autopsy programs to obtain high-quality metastatic tissue for analysis [15]. However, this material can naturally only be used for research purposes, but not for biomarker detection for individualized treatment decisions. This makes blood-based assays crucially important to individualize management of prostate cancer [16]. Profiling of blood offers several practical advantages, including the minimally invasive nature of sample acquisition, relative ease of standardization of sampling protocols, and the ability to obtain repeated samples over time. For example, the presence of circulating tumor cells (CTCs) in peripheral blood is a prognostic biomarker and a measure of therapeutic response in patients with prostate cancer [17-20]. Novel microfluidic devices enhance CTC capture [21-23] and allow to establish a non-invasive measure of intratumoral AR signaling before and after hormonal therapy [24]. Furthermore, prospective studies have demonstrated that mRNA expression signatures from whole blood can be used to stratify patients with CRPC into high- and low-risk groups [25,26].

Another option represents the analysis of plasma DNA [27]. One approach is the identification of known alterations previously found in the resected tumors from the same patients in plasma DNA for monitoring purposes [28,29]. Furthermore, recurrent mutations can be identified in plasma DNA in a subset of patients with cancer [30-32]. Given that chromosomal copy number changes occur frequently in human cancer, we developed an approach allowing the mapping of tumor-specific copy number changes from plasma DNA employing array-CGH [33]. At the same time, massively parallel sequencing of plasma DNA from the maternal circulation is emerging to a clinical tool for the routine detection of fetal aneuploidy [34-36]. Using essentially the same approach, that is, next-generation sequencing from plasma, the detection of chromosomal alterations in the circulation of three patients with hepatocellular carcinoma and one patient with both breast and ovarian cancer [37] and from 10 patients with colorectal and breast cancer [38] was reported.

However, the costs of the aforementioned plasma sequencing studies necessary for detection of rearrangements were prohibitive for routine clinical implementation [37,38]. In addition, these approaches are very time-consuming. Previously it had been shown that whole-genome sequencing with a shallow sequencing depth of about 0.1x is sufficient for a robust and reliable analysis of copy number changes from single cells [39]. Hence, we developed a different whole-genome plasma sequencing approach employing a benchtop high-throughput sequencing instrument, that is, the Illumina MiSeq, which is based on the existing Solexa sequencing-by-synthesis chemistry, but has dramatically reduced run times compared to the Illumina HiSeq [40]. Using this instrument we performed whole-genome sequencing from plasma DNA and measured copy number from sequence read depth. We refer to this approach as plasma-Seq. Furthermore, we enriched 1.3 Mbp consisting of exonic sequences of 55 high-interest cancer genes and 38 introns of genes, where fusion breakpoints have been described and subjected the DNA to next-generation sequencing at high coverage (approximately 50x). Here we present the implementation of our approach with 25 plasma samples from individuals without cancer and results obtained with whole genome sequencing of 13 plasma DNA samples derived from nine patients (five CRPC, four CSPC) with prostate cancer.

## Methods

### Patient eligibility criteria

This study was conducted among men with prostate cancer (Clinical data in Additional file 1, Table S1) who met the following criteria: histologically-proven, based on a biopsy, metastasized prostate cancer. We distinguished between CRPC and CSPC based on the guidelines on prostate cancer from the European Association of Urology [41], that is: 1, castrate serum levels of testosterone (testosterone <50 ng/dL or <1.7 nmol/L); 2, three consecutive rises of PSA, 1 week apart, resulting in two 50% increases over the nadir, with a PSA >2 ng/mL; 3, anti-androgen withdrawal for at least 4 weeks for flutamide and for at least 6 weeks for bicalutamide; 4, PSA progression, despite consecutive hormonal manipulations. Furthermore, we focused on patients who had ≥5 CTCs per 7.5 mL [19] and/or a biphasic plasma DNA size distribution as described previously by us [33].

The study was approved by the ethics committee of the Medical University of Graz (approval numbers 21-228 ex 09/10, prostate cancer, and 23-250 ex 10/11, prenatal plasma DNA analyses), conducted according to the Declaration of Helsinki, and written informed consent was obtained from all patients and healthy blood donors. Blood from prostate cancer patients and from male controls without malignant disease was obtained from the Department of Urology or the Division of Clinical Oncology, Department of Internal Medicine, at the Medical University of Graz. From prostate cancer patients we obtained a buccal swab in addition. Blood samples from pregnant females and from female controls without malignant disease were collected at the Department of Obstetrics and Gynecology, Medical University of Graz. The blood samples from the pregnant females were taken prior to an invasive prenatal diagnostic procedure.

### Plasma DNA preparation

Plasma DNA was prepared using the QIAamp DNA Blood Mini Kit (Qiagen, Hilden, Germany) as previously described [33]. Samples selected for sequence library construction were analyzed by using the Bioanalyzer instrument (Agilent Technologies, Santa Clara, CA, USA) to observe the plasma DNA size distribution. In this study we included samples with a biphasic plasma DNA size distribution as previously described [33].

### Enumeration of CTCs

We performed CTC enumeration using the automated and FDA approved CellSearch assay. Blood samples (7.5 mL each) were collected into CellSave tubes (Veridex, Raritan, NJ, USA). The Epithelial Cell Kit (Veridex) was applied for CTC enrichment and enumeration with the CellSearch system as described previously [42,43].

### Array-CGH

Array-CGH was carried out using a genome-wide oligonucleotide microarray platform (Human genome CGH 60K microarray kit, Agilent Technologies, Santa Clara, CA, USA), following the manufacturer's instructions (protocol version 6.0) as described [33]. Evaluation was done based on our previously published algorithm [33,44,45].

### HT29 dilution series

Sensitivity of our plasma-Seq approach was determined using serial dilutions of DNA from HT29 cell line (50%, 20%, 15%, 10%, 5%, 1%, and 0%) in the background of normal DNA (Human Genomic DNA: Female; Promega, Fitchburg, WI, USA). Since quantification using absorption or fluorescence absorption is often not reliable we used quantitative PCR to determine the amount of amplifiable DNA and normalized the samples to a standard concentration using the Type-it CNV SYBR Green PCR Kits (Qiagen, Hilden, Germany). Dilution samples were then fragmented using the Covaris S220 System (Covaris, Woburn, MA, USA) to a maximum of 150-250 bp and 10 ng of each dilution were used for library preparation to simulate plasma DNA condition.

### Plasma-Seq

Shotgun libraries were prepared using the TruSeq DNA LT Sample preparation Kit (Illumina, San Diego, CA, USA) following the manufacturer´s instructions with three exceptions. First, due to limited amounts of plasma DNA samples we used 5-10 ng of input DNA. Second, we omitted the fragmentation step since the size distribution of the plasma DNA samples was analyzed on a Bioanalyzer High Sensitivity Chip (Agilent Technologies, Santa Clara, CA, USA) and all samples showed an enrichment of fragments in the range of 160 to 340 bp. Third, for selective amplification of the library fragments that have adapter molecules on both ends we used 20-25 PCR cycles. Four libraries were pooled equimolarily and sequenced on an Illumina MiSeq (Illumina, San Diego, CA, USA).

The MiSeq instrument was prepared following routine procedures. The run was initiated for 1x150 bases plus 1x25 bases of SBS sequencing, including on-board clustering and paired-end preparation, the sequencing of the respective barcode indices and analysis. On the completion of the run, data were base called and demultiplexed on the instrument (provided as Illumina FASTQ 1.8 files, Phred+33 encoding). FASTQ format files in Illumina 1.8 format were considered for downstream analysis.

### Calculation of segments with identical log_2 _ratio values

We employed a previously published algorithm [46] to create a reference sequence. The pseudo-autosomal region (PAR) on the Y chromosome was masked and the mappability of each genomic position examined by creating virtual 150 bp reads for each position in the PAR-masked genome. Virtual sequences were mapped to the PAR-masked genome and mappable reads were extracted. Fifty thousand genomic windows were created (mean size, 56,344 bp) each having the same amount of mappable positions.

Low-coverage whole-genome sequencing reads were mapped to the PAR-masked genome and reads in different windows were counted and normalized by the total amount of reads. We further normalized read counts according to the GC content using LOWESS-statistics. In order to avoid position effects we normalized the sequencing data with GC-normalized read counts of plasma DNA of our healthy controls and calculated log_2 _ratios.

Resulting normalized ratios were segmented using circular binary segmentation (CBS) [47] and GLAD [48] by applying the CGHweb [49] framework in R [50]. These segments were used for calculation of the segmental z-scores by adding GC-corrected read-count ratios (read-counts in window divided by mean read-count) of all the windows in a segment. Z-scores were calculated by subtracting mean sum of GC-corrected read-count ratios of individuals without cancer (10 for men and 9 for women) of same sex and dividing by their standard-deviation.

$$z_{segments}=\frac{\sum ratio_{GC-corr}-mean\left(\sum ratio_{GC-corr,controls}\right)}{SD\sum \left(ratio_{GC-corr,controls}\right)}$$

### Calculation of z-scores for specific regions

In order to check for the copy-number status of genes previously implicated in prostate-cancer initiation or progression we applied z-score statistics for each region focusing on specific targets (mainly genes) of variable length within the genome. At first we counted high-quality alignments against the PAR-masked hg19 genome within genes for each sample and normalized by expected read counts.

$$ratio=\frac{reads_{region}}{reads_{expected}}$$

Here expected reads are calculated as

$$reads_{expected}=\frac{length_{region}}{length_{genome}}*reads_{total}$$

Then we subtracted the mean ratio of a group of controls and divided it by the standard deviation of that group.

$$z_{region}=\frac{ratio_{sample}-mean\left(ratio_{controls}\right)}{SD\left(ratio_{controls}\right)}$$

### Calculation of genome-wide z-scores

In order to establish a genome-wide z-score to detect aberrant genomic content in plasma, we divided the genome into equally-sized regions of 1 Mbp length and calculated z-scores therein.

Under the condition that all ratios were drawn from the same normal distribution, z-scores are distributed proportionally to Student's *t*-distribution with *n*-1 degrees of freedom. For controls, z-scores were calculated using cross-validation. In brief, z-score calculation of one control is based on means and standard deviation of the remaining controls. This prevents controls from serving as their own controls.

The variance of these cross-validated z-scores of controls is slightly higher than the variance of z-scores of tumor patients. Thus ROC performance is underestimated. This was confirmed in the simulation experiment described below.

In order to summarize the information about high or low z-score that was observed in many tumor patients squared z-scores were summed up.

$$S=\sum_{i\;from\;all\;Windows}z_{i}^{2}$$

Genome-wide z-scores were calculated from S-scores. Other methods of aggregation of z-score information, such as sums of absolute values or PA scores [38], performed poorer and were therefore not considered. Per window z-scores were clustered hierarchically by the *hclust *function of R using Manhattan distance that summed up the distance of each window.

In order to validate the diagnostic performance of the genome-wide z-score *in silico*, artificial cases and controls were simulated from mean and standard deviations of ratios from 10 healthy controls according to a normal distribution. Simulated tumor cases were obtained through multiplication of the mean by the empirical copy number ratio of 204 prostate cancer cases [9]. Segmented DNA-copy-number data were obtained via the cBio Cancer Genomics Portal [51].

To test the specificity of our approach at varying tumor DNA content, we performed *in-silico *dilutions of simulated tumor data. To this end we decreased the tumor signal using the formula below, where λ is the ratio of tumor DNA to normal DNA:

$$\left(\text{1}-\lambda \right)+\lambda \cdot ratio_{segment}$$

We performed ROC analyses of 500 simulated controls and 102 published prostate tumor data and their respective dilutions using the pROC R-package [52]. The prostate tumor data were derived from a previously published dataset [9] and the 102 cases were selected based on their copy number profiles.

### Gene-Breakpoint Panel: target enrichment of cancer genes, alignment and SNP-calling, SNP-calling results

We enriched 1.3 Mbp of seven plasma DNAs (four CRPC cases, CRPC1-3 and CRPC5; three CSPC cases, CSPC1-2 and CSPC4) including exonic sequences of 55 cancer genes and 38 introns of 18 genes, where fusion breakpoints have been described using Sure Select Custom DNA Kit (Agilent, Santa Clara, CA, USA) following the manufacturer's recommendations. Since we had very low amounts of input DNA we increased the number of cycles in the enrichment PCR to 20. Six libraries were pooled equimolarily and sequenced on an Illumina MiSeq (Illumina, San Diego, CA, USA).

We generated a mean of 7.78 million reads (range, 3.62-14.96 million), 150 bp paired-end reads on an Illumina MiSeq (Illumina, San Diego, CA, USA). Sequences were aligned using BWA [53] and duplicates were marked using picard [54]. We subsequently performed realigning around known indels and applied the Unified Genotyper SNP-calling software provided by the GATK [55].

We further annotated resulting SNPs by employing annovar [56] and reduced the SNP call set by removing synonymous variants, variants in segmental duplications and variants listed in the 1000 Genome Project [57] and Exome sequencing (Project Exome Variant Server, NHLBI Exome Sequencing Project (ESP), Seattle, WA) [58] with allele frequency >0.01.

We set very stringent criteria to reduce false positives according to previously published values [37]: a mutation had to be absent from the constitutional DNA sequencing and the sequencing depth for the particular nucleotide position had to be >20-fold. Furthermore, all putative mutations or breakpoint spanning regions were verified by Sanger sequencing.

### Split-read analysis

Since plasma DNA is fragmented the read pair method is not suitable for identification of structural rearrangements [59] and therefore we performed split-read analysis of 150 bp reads. We used the first and the last 60 bp of each read (leaving a gap of 30 bp) and mapped these independently. We further analyzed discordantly mapped split-reads by focusing on targeted regions and filtering out split-reads mapping within repetitive regions and alignments having a low mapping quality (<25). Reads where discordantly mapped reads were found were aligned to the human genome using BLAT [60] to further specify putative breakpoints.

### Data deposition

All sequencing raw data were deposited at the European Genome-phenome Archive (EGA) [61], which is hosted by the EBI, under accession numbers EGAS00001000451 (Plasma-Seq) and EGAS00001000453 (Gene-Breakpoint Panel).

## Results

### Implementation of our approach

Previously, we demonstrated that tumor-specific, somatic chromosomal alterations can be detected from plasma of patients with cancer using array-CGH [33]. In order to extend our method to a next-generation sequencing-based approach, that is, plasma-Seq, on a benchtop Illumina MiSeq instrument, we first analyzed plasma DNA from 10 men (M1 to M10) and nine women (F1 to F9) without malignant disease. On average we obtained 3.3 million reads per sample (range, 1.9-5.8 million; see Additional file 1, Table S2) and applied a number of filtering steps to remove sources of variation and to remove known GC bias effects [62-64] (for details see Material and Methods).

**Figure 1:**
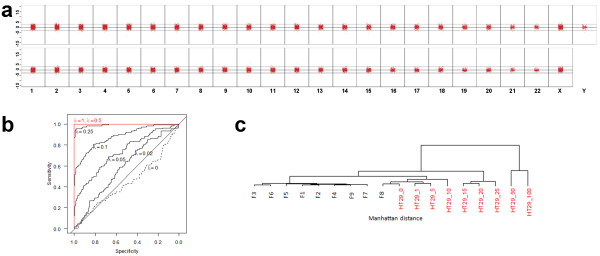
**Implementation of our approach using plasma DNA samples from individuals without cancer and simulations**. (**a**) Z-scores calculated for sequential 1-Mbp windows for 10 male (upper panel) and 9 female (lower panel) individuals without malignant disease. (**b**) Detection of tumor DNA in plasma from patients with prostate cancer using simulated copy-number analyses. ROC analyses of simulated mixtures of prostate cancer DNA with normal plasma DNA using the genome-wide z-score. Detection of 10% circulating tumor DNA could be achieved with a sensitivity of >80% and specificity of >80%. (c) Hierarchical cluster analysis (Manhattan distances of chromosomal z-scores) with normal female controls and the HT29 serial dilution series. One percent of tumor DNA still had an increased genome-wide z-score and did not cluster together with the controls (for details see text).

We performed sequential analyses of 1-Mbp windows (*n*=2,909 for men; *n*=2,895 for women) throughout the genome and calculated for each 1-Mbp window the z-score by cross-validating each window against the other control samples from the same sex (details in Material and Methods). We defined a significant change in the regional representation of plasma DNA as >3 SDs from the mean representation of the other healthy controls for the corresponding 1-Mbp window. A mean of 98.5% of the sequenced 1-Mbp windows from the 19 normal plasma samples showed normal representations in plasma (Figure 1a). The variation among the normalized proportions of each 1-Mbp window in the plasma from normal individuals was very low (average, 47 windows had a z-score £-3 or ≥3; range of SD, ±52%) (Figure 1a).

In addition, we calculated 'segmental z-scores' where the z-scores are not calculated for 1-Mbp windows but for chromosomal segments with identical copy number. In order to determine such segments we employed an algorithm for the assignment of segments with identical log_2 _ratios [39,46] (Material and Methods) and calculated a z-score for each of these segments (hence, 'segmental z-scores'). As sequencing analyses of chromosome content in the maternal circulation are now frequently being used for detection of fetal aneuploidy [34,36] and as our mean sequencing depth is lower compared to previous studies, we wanted to test whether our approach would be feasible for this application. To this end we obtained two plasma samples each of pregnancies with euploid and trisomy 21 fetuses and one each of pregnancies with trisomies of chromosomes 13 and 18, respectively. In the trisomy cases the respective chromosomes were identified as segments with elevated log_2 _ratios and accordingly also increased z-scores (Additional file 2).

### Sensitivity and specificity of our approach

We wanted to gain insight into the sensitivity of our approach to detect tumor-derived sequences in a patient's plasma. To this end we calculated a genome-wide z-score for each sample (Material and Methods). The main purpose of the genome-wide z-score is to distinguish between aneuploid and euploid plasma samples. The genome-wide z-score from the plasma of male individuals ranged from -1.10 to 2.78 and for female individuals from -0.48 to 2.64. We performed receiver operating characteristic (ROC) analyses of simulated next-generation sequencing data from 102 published prostate cancer data and 500 simulated controls based on the data from our healthy individuals. Using the equivalent of one-quarter MiSeq run, these analyses suggested that using the genome-wide z-score tumor DNA concentrations at levels ≥10% could be detected in the circulation of patients with prostate cancers with a sensitivity of >80% and specificity of >80% (Figure 1c).

To test these estimates with actual data we fragmented DNA from the colorectal cancer cell line HT29 to sizes of approximately 150-250 bp to reflect the degree of fragmented DNA in plasma and performed serial dilution experiments with the fragmented DNA (that is, 50%, 20%, 15%, 10%, 5%, 1%, and 0%). We established the copy-number status of this cell line with undiluted, that is, 100%, DNA using both array-CGH and our next-generation sequencing approach (Additional file 3) and confirmed previously reported copy number changes [65,66]. Calculating the genome-wide z-score for each dilution we noted its expected decrease with increasing dilution. Whereas the genome-wide z- score was 429.74 for undiluted HT29 DNA, it decreased to 7.75 for 1% (Additional file 1, Table S2). Furthermore, when we performed hierarchical cluster analysis the female controls were separated from the various HT29 dilutions, further confirming that our approach may indicate aneuploidy in the presence of 1% circulating tumor DNA (Figure 1d).

### Plasma analysis from patients with cancer

**Figure 2:**
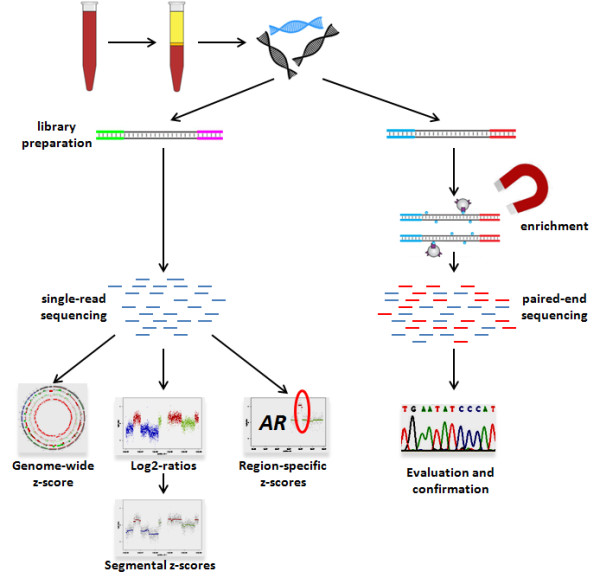
**Outline of our whole-genome plasma analysis strategy**. After blood draw, plasma preparation, and DNA-isolation we start our analysis, which is two-fold: first (left side of the panel), an Illumina shotgun library is prepared (time required, approximately 24 h). Single-read whole genome plasma sequencing is performed with a shallow sequencing depth of approximately 0.1x (approximately 12 h). After alignment we calculate several z-scores: a genome-wide z-score, segments with identical log2-ratios required to establish corresponding segmental z-scores, and gene-specific z-scores, for example, for the *AR*-gene. Each of these z-scores calculations takes approximately 2 h so that these analyses are completed within 48 h and the material costs are only approximately €300. Second (right side of the panel), we prepare a library using the SureSelect Kit (Agilent) and perform sequence enrichment with our GB-panel (approximately 48-72 h), consisting of 55 high-interest genes and 38 introns with frequent fusion breakpoints. The GB-panel is sequenced by paired-end sequencing with an approximately 50x coverage (around 26 h). The evaluation of the sequencing results may take several hours, the confirmation by Sanger sequencing several days. Hence, complete analysis of the entire GB-panel analysis will normally require around 7 days.

Our analysis of plasma samples from patients with cancer is two-fold (Figure 2): (a) we used plasma-Seq to calculate the genome-wide z-score as a general measure for aneuploidy and the segmental z-scores to establish a genome-wide copy number profile. The calculation of the segments with identical log_2 _ratios takes only 1 h and also provides a first assessment of potential copy number changes. Calculation of the z-scores for all segments and thus definite determination of over- and under-represented regions requires about 24 h. (b) In addition, we sequenced with high coverage (approximately 50x) 55 genes frequently mutated in cancer according to the COSMIC [67] and Cancer Gene Census [68] databases (Additional file 1, Table S3), and 38 introns often involved in structural somatic rearrangements, including recurrent gene fusions involving members of the E twenty-six (ETS) family of transcription factors to test for *TMPRSS2-ERG*-positive prostate cancers (herein referred to as GB-panel (Gene-Breakpoint panel)). In a further step identified mutations were verified by Sanger sequencing from both plasma DNA and constitutional DNA (obtained from a buccal swab) to distinguish between somatic and germline mutations. If needed, somatic mutations can then be used to estimate by deep sequencing the fraction of mutated tumor DNA in the plasma.

### Plasma-Seq and GB-panel of patients with prostate cancer

We then obtained 13 plasma samples from nine patients with metastatic prostate cancer (five with castration-resistant disease, CRPC1 to CRPC5, and four with castration-sensitive disease, CSPC1 to CSPC4. Furthermore, from each of patients CRPC1 and CSPC1 we obtained three samples at different time points (Clinical data in Additional file 1, Table S1). Applying plasma-Seq, we obtained on average 3.2 million reads (range, 1.1 (CSPC4) to 5.2 (CRPC5) million reads) for the plasma samples from patients with prostate cancer per sample (see Additional file 1, Table S2).

**Figure 3:**
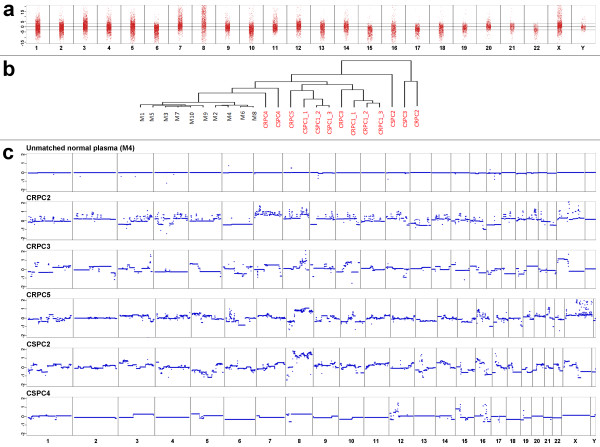
**Copy number analyses of plasma samples from men with prostate cancer**. (**a**) Z-scores calculated for 1-Mbp windows from the 13 plasma samples of patients with prostate cancer showed a high variability (compare with same calculations from men without malignant disease in Figure 1a, upper panel). (**b**) Hierarchical clustering (Manhattan distances of chromosomal z-scores) separates samples from men without cancer and with prostate cancer. (**c**) Copy number analyses, based on segmental z-scores, of an unmatched normal male plasma sample and five plasma samples from patients with prostate cancer (CRPC2, CRPC3, CRPC5, CSPC2, and CSPC4). The Y-axis indicates log_2_-ratios.

To assess whether plasma-Seq allows discrimination between plasma samples from healthy men and men with prostate cancer we first calculated the genome-wide z-score. In contrast to the male controls (Figure 1a), the 1-Mbp window z-scores showed a substantial variability (Figure 3a) and only a mean of 79.3% of the sequenced 1-Mbp windows from the 13 plasma samples showed normal representations in plasma in contrast to 99.0% of the cross-validated z-scores in the sample of controls (*P*=0.00007, Wilcoxon test on sample percentages). Accordingly, the genome-wide z-score was elevated for all prostate cancer patients and ranged from 125.14 (CRPC4) to 1155.77 (CSPC2) (see Additional file 1, Table S2). Furthermore, when we performed hierarchical clustering the normal samples were separated from the tumor samples (Figure 3b), suggesting that we can indeed distinguish plasma samples from individuals without malignant disease from those with prostate cancer.

Applying the GB-panel, we achieved on average a coverage of ≥50x for 71.8% of target sequence (range, 67.3% (CSPC4) to 77.6% (CSPC2)) (see Additional file 1, Table S4). Using very stringent conditions (see Material & Methods) the GB-panel allowed us to identify 12 mutations in all seven patients for which the analyses were performed (that is, CRPC1-3, CRPC5, CSPC1-2, and CSPC4). Sanger sequencing confirmed the presence of five of these mutations in both plasma and the respective constitutional DNA, whereas seven mutations were only confirmed in plasma but not in the constitutional DNA. The latter mutations, which were observed in five patients (that is, CRPC2-3, CRPC5, CSPC2, CSPC4), are likely somatic mutations and occurred in genes previously implicated in prostate cancer tumorigenesis, such as *TP53*, *BRCA1*, *BRCA2*, and *MLL3 *(see Additional file 1, Table S4). We used these somatic mutations for ultra-deep sequencing with an average coverage of 362,016 (range, 307,592 to 485,467) to estimate the tumor fraction. Using these estimates the tumor fraction was lowest in CSPC4 with 30.75% and highest in CRPC5 with 54.49%.

Plasma-Seq from these patients exhibited a wide range of copy number aberrations indicative of malignant origin, including those that have been previously reported in prostate tumors. For example, the three CRPC patients (that is, CRPC2-3, CRPC5) had high-level gains in a region on chromosome × including the *AR *locus. Over-representation of 8q regions was observed in all five patients and loss of 8p regions in three patients (CRPC5, CSPC2, and CSPC4) (Figure 3c).

As control we performed array-CGH analyses of all plasma cases as described [33] in parallel (see Additional file 4). These array-CGH profiles had a great concordance with those obtained with plasma-Seq.

### *TMPRSS2-ERG *fusion mapping

The fusion through deletion *TMPRSS2-ERG *rearrangement results in a well-defined 3-Mbp interstitial deletion on chromosome 21 [69,70] and occurs in approximately 50% of prostate cancer cases [71]. We tested whether our approach would allow distinguishing *TMPRSS2-ERG*-positive from *TMPRSS2-ERG*-negative prostate cancers.

**Figure 4:**
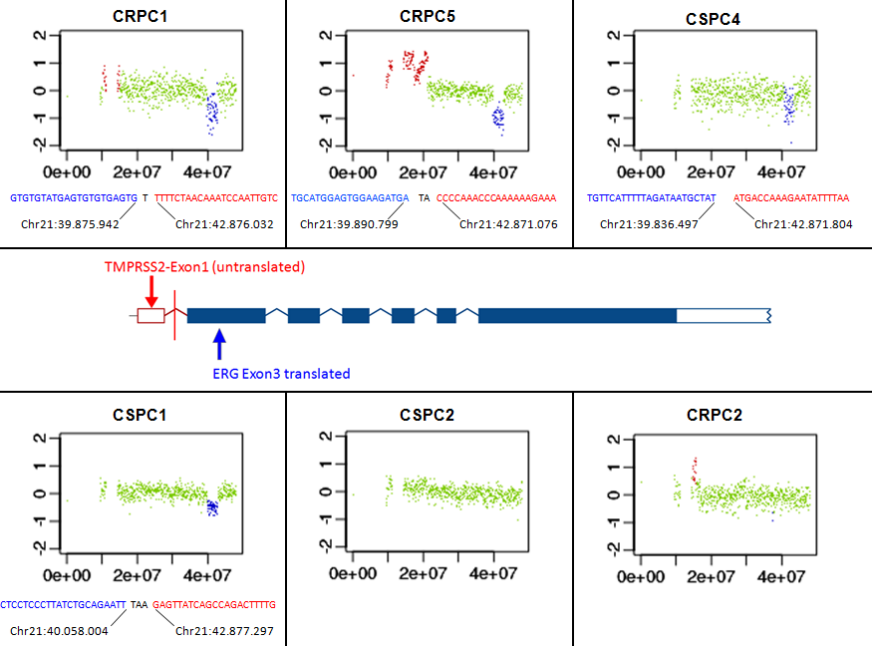
**Identification of the *TMPRSS2-ERG *associated 3-Mb deletion on chromosome 21 and mapping of the breakpoints**. Exemplary log_2 _ratio plots of chromosome 21 from plasma DNA of several patients (regions with log_2 _ratios >0.2 are shown in red and those with log_2 _ratios <-0.2 in blue). A deletion with size of 3 Mbp located at the *TMPRSS2-ERG *region was visible in patients CRPC1, CRPC5, CSPC4, and CSPC1. For comparison we also included chromosome 21 plots from CSPC2 and CRPC2 without this deletion. Mapping of the exact breakpoints was based on fusion transcripts identified with our GB-panel. In CRPC1, CRPC5, and CSPC4 the breakpoints were in exon 1 of the *TMPRSS2 *gene and exon 3 of the *ERG *gene, respectively (center panel). In CSPC1 the proximal breakpoint was approximately 24 Kb upstream of the *ERG *gene.

Plasma-Seq identified a 3-Mbp deletion at the *TMPRSS2-ERG *location on chromosome 21 in five patients (CRPC1, CRPC3, CRPC5, CSPC1, and CSPC4) (Figure 4). To further confirm the presence of the deletions we analyzed the sequences obtained with the GB-panel with the split-read method (see Material and Methods). We identified several fusion spanning reads in each of the aforementioned patients (see Additional file 1, Table S4), which enabled us to map the breakpoints with bp resolution (Figure 4). Most of our deletions originate from exon 1 of *TMPRSS2 *and are fused to exon 3 of *ERG *consistent with previous reports [71]. We then further confirmed all *TMPRSS2-ERG *fusions with Sanger sequencing (data not shown).

### Analyses of serial plasma samples

**Figure 5:**
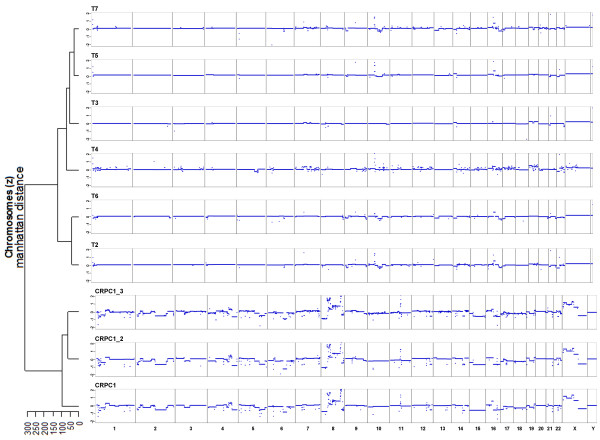
**Analyses of tumor and serial plasma samples from patient CRPC1**. DNA was extracted from six different regions (designated as T2-T7) from the primary tumor and separately analyzed by our whole-genome sequencing approach (corresponding histology images are in Additional file 5). The first plasma sample (CRPC1) was obtained 13 years after resection of the primary tumor, the interval between the first and second (CRPC1_2) sample was 7 months and between the second and third (CRPC1_3) 2 months. The patient had stable disease under AD and chemotherapy. Hierarchical clustering (Manhattan distances of chromosomal z-scores) of the plasma samples and the sectors of the primary tumor is shown on the left side, the samples are shown in the corresponding order.

We had the opportunity to perform serial plasma analyses from two patients: CRPC1 and CSPC1. CRPC1 had his primary tumor completely resected in 1999, (13 years before we performed our plasma analyses). Since the primary tumor appeared to be very heterogeneous (see Additional file 5) pathologist-guided dissection was carefully performed from six different regions (designated as T2-T7). We performed our whole-genome sequencing analysis for each region separately and found different copy-number changes in each sector. Common changes included partial gain of 16p (observed in T2, T4, T5, T6, and T7) and partial losses of 10q (T2, T6, T5, and T7), 13q (T2, T6, and T7), and 16q (T2, T5, T6, and T7) (Figure 5). These various findings in different tumor sectors are consistent with a multifocal disease, which is frequently encountered in prostate cancer [16].

Plasma samples were taken at three different time points over a 9-month period (we refer to them in addition to CRPC1 as CRPC1_2 and CRPC1_3). At the time of our plasma collections the patient was castration resistant and had stable disease under ongoing ADT and chemotherapy. Plasma-Seq identified again multiple prostate cancer-associated chromosomal alterations, such as 8p loss, gain of 8q regions, the 3-Mbp *TMPRSS2-ERG *deletion on chromosome 21, and *AR *amplification (Figures 4 and 5). Thus, plasma-Seq identified multiple rearrangements, that is, the *TMPRSS2-ERG *deletion on chromosome 21, which had not been present in the primary tumor. Furthermore, plasma-Seq yielded remarkably similar results in our three analyses over the 9-month period (Figure 5), which is in agreement with the clinically stable disease and suggests the presence of one dominant clone releasing DNA into the circulation. This is consistent with the proposed monoclonal origin of metastatic prostate cancer [8]. Hierarchical clustering confirmed the concordance between the three plasma-Seq copy number profiles and the tremendous differences to the various sectors of the primary tumor (Figure 5).

**Figure 6:**
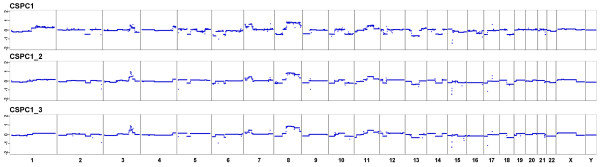
**Analyses of serial plasma samples from patient CSPC1**. The first plasma sample (CSPC1) was collected 12 months after initial diagnosis, only a biopsy had been taken from the primary tumor to confirm diagnosis. The interval between the first and second (CSPC1_2) sample was 5 months and 1 month between the second and third (CSPC1_3). The patient was clinically responding to castration therapy.

We collected the first plasma sample from CSPC1 about 12 months after initial diagnosis and two other samples over a 6-month period (CSPC1, CSPC1_2, and CSPC1_3). Only a biopsy had been taken from the primary tumor to confirm diagnosis. The patient was clinically responding to castration therapy. We observed again a number of copy number changes, many of those characteristic of prostate cancer (Figure 6), such as the *TMPRSS2-ERG *deletion (Figure 4). There was no *AR *amplification as expected for a CSPC case.

The high similarity of copy number changes at various time points is another confirmation of the high reliability and robustness of our approach.

### Evaluation of copy number changes of prostate cancer genes

The evaluation of 1-Mbp or segmental z-scores each involves relatively large regions. We wanted to test whether z-scores can also be calculated for much smaller regions, that is specific genes, and calculated gene-specific z-scores (see Material & Methods).

For example, in prostate cancer, one of the most interesting regions is the *AR*-locus on chromosome Xq12, which is amplified in approximately 33% of patients with CRPC [72]. As expected, none of the male healthy controls had an amplification of *AR*, whereas *AR *amplification was present in four of the five CRPC cases. In order to validate the plasma-Seq gene-specific copy number estimates with another approach we selected a subset of samples (CRPC1, CRPC2, CRPC5, CSPC1, CSPC1_2, and CSPC2) for validation of the *AR *copy-number status with qPCR. In fact, we observed a very close correlation between the plasma-Seq and the qPCR values (see Additional file 6). Interestingly, CRPC1 had only a duplication of the *AR *region and the *AR *copy number did not change over our observation period of 9 months, which was consistent with the clinically stable disease. One of the CSPC cases, CSPC4, had a slightly increased *AR *ratio (ratio, 1.46; z-score, 4.60). Whether such a value may indicate the beginning of ADT resistance remains presently unclear, as sufficient follow-up data were not available.

We also tested our approach for some other genes, which have frequently been implicated in prostate cancer. For example, evidence for cooperation between *AR *and *NCOA2 *amplifications on 8q13.3 in early prostate cancer was reported [73]. However, alternatively it was suggested that tumors first acquire *NCOA2 *amplification along with broad amplifications on chromosome 8q [6]. Our gene-specific z-score identified *NCOA2 *gene amplifications in five patients (CRPC1, CRPC5, CSPC1-3), thus, two CRPC and three CSPC cases, which may support the notion that *NCOA2 *amplifications may occur prior to *AR *amplification [6].

Loss of *PTEN *on 10q23.31 occurs in approximately 40% of prostate cancers [9,74]. We observed *PTEN *loss in five patients (CRPC3-5, CSPC1, and CSPC3); that is, in three CRPC and two CSPC cases. The AKT-inactivating phosphatase *PHLPP1 *on 18q21.33 has recently been identified as a prostate tumor suppressor [75]. We found that this gene was lost in four patients (CRPC1, CRPC3, CSPC1-2); that is, in two CRPC and two CSPC cases. Furthermore, it has recently been reported that the *TMPRSS2-ERG *fusion is associated with a deletion at chromosome 3p14 that includes the *FOXP1 *gene [9]. In fact, we observed loss of this region in five of our patients (CRPC1-2, CRPC4, CSPC1, CSPC4) and four of these patients (CRPC1-2, CSPC1, CSPC4) did indeed have the *TMPRSS2-ERG *fusion, confirming the association between these two loci.

In summary, our results suggest that gene-specific information can be derived from plasma-Seq, which may facilitate the evaluation of pathways potentially comprised in prostate cancer.

## Discussion

This study represents the first whole-genome sequencing analysis from plasma DNA of patients with prostate cancer. Usually the identification of tumor genotypes that inform selection of targeted therapies is performed on the initial diagnostic specimen. However, these may not be readily available or in case of fine needle aspirates not sufficient for molecular analyses, as was the case for our patients who presented with metastatic disease. The only exception in our cohort was CRPC1, who had recurrent disease many years after initial operative treatment. We could demonstrate that the initial primary tumor specimen represented multifocal disease and none of the analyzed sectors was representative of the metastatic clone, which arose 13 years later. Thus, molecular analysis of plasma may provide a non-invasive approach for tumor cell genotyping, which can easily be repeated during the course of therapy.

Multiple lines of evidence support the copy number changes observed. First, the observation of known prostate cancer alterations in our dataset indicates successful performance of our assay. Second, our previously published array-based plasma method [33] was applied in parallel to confirm the copy number aberrations observed with plasma-Seq. Third, we identified the well characterized 3-Mbp interstitial 21q22.2-3 deletion spanning *ERG *and *TMPRSS2 *on chromosome 21 [69,70] and confirmed its presence with our GB-panel and Sanger sequencing. Fourth, for two of our patients we were able to repeat our analysis at different time points. These repeated analyses revealed a high degree of similarity among samples from the same patient. The shared copy number aberrations were indicative of common lineage, which is consistent with the view that metastases in this disease are of monoclonal origin [8]. Finally, implementation of our approach with 19 samples from individuals without cancer and five plasma samples from pregnant females with aneuploidy fetuses further confirmed the reliability and robustness of our approach.

Tests for sensitivity and specificity of our approach suggested that tumor DNA concentrations at levels ≥10% can be detected with a sensitivity of >80% and specificity of 80%. Furthermore, our simulations and HT29 dilution experiments suggested that the genome-wide z-score detects aneuploidy even at tumor DNA concentrations of only 1%. In general, the resolution of non-invasive tumor genome-wide scans from plasma is limited by the depth of the sequencing and the percentage of tumor fragments in the plasma. Therefore, previously published similar studies [37,38] employed high-throughput sequencing platforms tailored chiefly toward large-scale applications. As a consequence, footprints, workflows, reagent costs, and run times are poorly matched to the needs of small laboratories and furthermore, the cost of the sequencing necessary for detection of rearrangements at this level is prohibitive for routine clinical implementation [38]. In contrast, advantages of a benchtop high-throughput sequencing instrument include the speed of analyses and the reduced costs. A MiSeq run produces a throughput of 1.6 Gbp with a read length of 150 bp [40]. As whole-genome sequencing with a 0.1x coverage was reported to yield robust and reliable copy-number measurements from single cells [39], we tested such a sequencing approach for our plasma analyses. Accordingly, we found that the characteristics of the MiSeq are sufficient for our plasma-Seq purposes. Especially attractive features of this strategy include the speed (library prep, approximately 24 h; sequencing of 150 bp single reads, approximately 12 h; identification of segments with identical log_2 _ratios, approximately 2 h; calculation of z-scores, 30 min) and the costs (approximately €300) with which the aneuploidy scoring by plasma-Seq can be performed. In contrast, completion of the GB-panel analysis, done at 50x coverage, will normally require at least 7 days (library prep, approximately 24 h; targeted enrichment, approximately 48-72 h; sequencing 150 bp paired end, approximately 26 h; evaluation and SNP calling, several hours) not including verifications of mutations by Sanger sequencing or estimation of the fractional load of tumor fragments by deep sequencing.

A disadvantage of low coverage whole-genome sequencing is that structural inter- and intrachromosomal rearrangements cannot be identified with high confidence. This is because plasma DNA fragments, whose paired-end reads map to different chromosomes or to the same chromosome but at large distances (several kb) apart, will likely not be detected in multiple reads. Another disadvantage is the reduced resolution for identification of mutations. However, several large scale whole-exome or whole-genome sequencing studies consistently reported low overall mutation rates even in heavily treated CRPCs ranging from 0.9 to 2.00 mutations per mega base [9,11-14]. These studies confirmed that the most commonly mutated gene was *AR*, however no single gene other than *AR *had frequent mutations and even common, broadly mutated oncogenes such as *PIK3CA*, *KRAS*, and *BRAF *are not commonly mutated in prostate cancer [9]. We addressed both issues, structural rearrangements and mutations, with a focused sequencing approach with higher coverage. Focused sequencing, such as our GB-panel, with tailored design and analytical prioritization strategies may represent an attractive alternative to large-scale whole-genome sequencing in terms of speed and costs. Such a focused approach is flexible and can easily be adapted if new, important genes or regions evolve from large-scale sequencing projects.

Another potential short-coming is that we do not know whether the changes observed in the plasma are related to the primary tumor or to any of the metastatic sites. In fact, it is currently unknown whether all tumor cells contribute to the plasma DNA equally and which factors influence the release of tumor DNA into the circulation. Further studies are needed to determine whether changes observed by plasma-Seq represent an average of the DNA alterations from all malignant sites or whether they show characteristic changes of the dominant tumor cell clone at the time of the blood collection.

At present we do not know how our plasma DNA signatures perform compared with other emerging candidate markers, for example, CTC analysis [24]. However, our approach circumvents an inherent limitation of all published CTC-based studies, that is, it is not focused on EpCAM-positive CTCs. Furthermore, plasma isolation does not necessitate special equipment as usually required for CTC isolation [21-23]. As we already have plasma-Seq data from patients with colon and breast cancer our method may also be applicable to other tumor types.

Whether these blood copy-number signatures will be true game changers for the management of prostate cancer has to be further evaluated. Drug development for castration-resistant prostate cancer is an area of intensive research and several new agents are currently being tested in phase 3 clinical trials. Interrogation of the genomic signature may reveal whether those targeted therapies are effectively hitting their target *in vivo*, thus providing information that may be useful in guiding therapeutic decisions.

## Conclusions

Our strategy may contribute to a better definition of the evolution towards a castration-resistant disease and could potentially aid in identifying patients more or less likely respond to AR-targeted therapies. The simplicity and the costs of our test are attractive and might ease the clinical translation. However, the extent to which these signatures contribute independent prognostic or predictive value beyond clinicopathological variables must be explored in more depth.

## Abbreviations

ADT: Androgen deprivation therapy; AR: Androgen receptor; BLAT: Basic Local Alignment Search Tool (BLAST)-like alignment tool; BWA: Burrows-Wheeler Aligner; CGH: Comparative genomic hybridization; CRPC: Castration-resistant prostate cancer; CSPC: Castration-sensitive prostate cancer; CTC: Circulating tumor cell; EBI: European Bioinformatics Institute; EGA: European Genome-Phenome Archive; ESP: Exome Sequencing Project; GATK: Genome Analysis Toolkit; GB-panel: Gene-breakpoint panel; ROC: Receiver-operating characteristic; SD: Standard-deviation.

## Competing interests

The authors declare that they have no competing interests.

## Authors' contributions

EH, PU, JB, TB, MA, and CP carried out the molecular genetic studies, sequencing and deep sequencing, and participated in the sequence alignment. SG, KF, MP, FE, MH, and HS contributed patient material and reviewed the clinical data. FQ, PU, and EH performed the statistical analysis. SM and GH reviewed the histology. SR and KP performed CTC identification and enumeration. EH, PU, JBG, and MRS participated in the design and coordination of the study, project planning, and drafted the manuscript. All authors read and approved the final manuscript.

## Supplementary Material

Additional file 1 **Supplementary tables 1-4**. Click here for file

Additional file 2 **Plasma DNA analyses from pregnant women**. Plasma DNA analyses from maternal blood with pregnancies with a trisomy 21 fetus (first panel), a trisomy 13 fetus (second panel), a trisomy 18 fetus (third panel), and an euploid fetus (fourth panel) (X-axis: Chromosome; Y-axis: z-score). Click here for file

Additional file 3 **Copy-number status of the HT29 cell line**. The upper panel illustrates the array-CGH profile, the lower panel the profile obtained with our next-generation sequencing approach. Both panels illustrate the copy number profile with undiluted, that is, 100%, DNA. In the array-CGH profile the multicolor bar codes at the top or bottom of the ratio profiles illustrate the results obtained during the iterative calculations with various window sizes, the single green and red bars summarize the regions which were gained or lost based on all calculations (for details see [44]). Black parts in the profile represent balanced regions, lost regions appear in red, and gained regions in green. Click here for file

Additional file 4 **Array-CGH evaluations as control for our plasma-Seq approach: Array-CGH profiles of plasma samples CRPC2, CRPC3, CRPC5, CSPC2, and CSPC4**. For all array-CGH profiles the multicolor bar codes at the top or bottom of the ratio profiles illustrate the results obtained during the iterative calculations with various window sizes, the single green and red bars summarize the regions which were gained or lost based on all calculations (for details see [44]). Black parts in the profile represent balanced regions, lost regions appear in red, and gained regions in green. Previously we had already demonstrated the use of array-CGH analyses for the analysis of plasma DNA [33]. The array-CGH profiles show a great concordance with those obtained with plasma-Seq. Click here for file

Additional file 5 Histology samples from the primary tumor of patient CRPC1. The six different samples are arranged according to the hierarchical clustering (Manhattan distances of chromosomal z-scores) from Figure 5, the corresponding part of the tree is shown to the left. From each sector the most common (left) and second most common (right) patterns are shown. Relating the hierarchical clustering of the chromosomal alterations in the various sectors of the primary tumor to morphological features, no clear picture emerges. Regarding the growth pattern, T2 and T3 seem closely related as are T4 and T5, which is not reflected in the clustering analysis of the chromosomal alterations. Based on nuclear staining features, T7 seems similar to T2 and T3. T6 also shares features of T2 and T3. Taking into account the changes detected in circulating DNA, the most likely explanation is a complex multifocal disease resulting in a complex morphological as well as genetic pattern. Click here for file

Additional file 6 Validation of the *AR *copy number status with qPCR for plasma samples CRPC1, CRPC2, CRPC5, CSPC1, CSPC1_2, and CSPC2 showing a very close correlation between the plasma-Seq and the qPCR values. Click here for file
