# Supplementary material for: Tumor-associated copy number changes in the circulation of patients with prostate cancer identified through whole-genome sequencing
Source: Genome Med. 2013 Apr 5;5(4):30. doi: 10.1186/gm434 (PMC3707016; doi:10.1186/gm434)
Supplement: Additional file 1 — Supplementary tables 1-4. [file gm434-S1.DOCX]

**Additional Files: Tables**

**Table S1**

**Patients’ characteristics and clinical data**

| Patient | Age at diagnosis | Interval  between diagnosis and sampling  (months) | pTNM or  Gleason score | PSA [ng/ml] | DNA conc. [ng/ml plasma] | No. of CTC |
| --- | --- | --- | --- | --- | --- | --- |
| CRPC1 | 69 | 153 | pT3aG3R1NxM0 | 444.1 | 39.9 | 150 |
| CRPC2 | 84 | 0 | 10 | 808.0 | 106.2 | 58 |
| CRPC3 | 63 | 80 | 9 | 34.7 | 11.5 | 18 |
| CRPC4 | 50 | 144 | NA | 452.7 | 5.5 | 3 |
| CRPC5 | 63 | 144 | NA | 593.9 | 51.6 | 105 |
| CSPC1 | 61 | 0 | 9 | 6471.0 | 17.1 | 27 |
| CSPC2 | 72 | 0 | 9 | 17.4 | 8.3 | 0 |
| CSPC3 | 72 | 0 | 10 | 394.7 | 3.6 | 140 |
| CSPC4 | 82 | 0 | 10 | 773.7 | 43.7 | 150 |

NA, not available

**Table S2**

**Summary of next-generation plasma sequencing (plasma-Seq) analyses**

| Sample | Patient diagnosis | Total bases^1^ | Total reads^2^ | Coverage^3^ | Genome-wide z-score^4^ |
| --- | --- | --- | --- | --- | --- |
| M1 | Control male | 424315445 | 2846648 | 0.14 | 2.78 |
| M2 | Control male | 490008446 | 3317609 | 0.16 | 1.42 |
| M3 | Control male | 358704014 | 2412673 | 0.12 | -0.87 |
| M4 | Control male | 294534884 | 1995096 | 0.10 | 0.98 |
| M5 | Control male | 415728206 | 2792389 | 0.13 | -0.60 |
| M6 | Control male | 317128667 | 2127400 | 0.10 | -0.13 |
| M7 | Control male | 405278682 | 2733847 | 0.13 | -1.10 |
| M8 | Control male | 296945684 | 2010569 | 0.10 | -0.77 |
| M9 | Control male | 419506909 | 2825164 | 0.14 | -0.18 |
| M10 | Control male | 261432529 | 1781771 | 0.08 | 0.74 |
| F1 | Control female | 456467502 | 3079634 | 0.15 | -0.40 |
| F2 | Control female | 369675975 | 2484554 | 0.12 | -0.45 |
| F3 | Control female | 442158682 | 2965444 | 0.14 | 0.01 |
| F4 | Control female | 484350661 | 3249872 | 0.16 | -0.47 |
| F4 | Control female | 556849888 | 3734647 | 0.18 | -0.27 |
| F6 | Control female | 753941007 | 5055021 | 0.24 | -0.24 |
| F7 | Control female | 403609282 | 2705935 | 0.13 | -0.34 |
| F8 | Control female | 821540354 | 5511091 | 0.27 | 2.64 |
| F9 | Control female | 346062153 | 2322371 | 0.11 | -0.48 |
| P1 | Pregnant female | 312087661 | 2094656 | 0.10 | 1.34 |
| P2 | Pregnant female | 217181695 | 1454190 | 0.07 | -0.07 |
| P3 | Pregnant female | 116660394 | 781990 | 0.04 | 0.67 |
| P4 | Pregnant female | 314792205 | 2106298 | 0.10 | 0.62 |
| CRPC1 | Castration-resistant | 173057476 | 1146076 | 0.06 | 381.48 |
| CRPC1_2 | Castration-resistant | 392809122 | 2660914 | 0.13 | 522.33 |
| CRPC1_3 | Castration-resistant | 662983919 | 4478846 | 0.21 | 436.51 |
| CRPC2 | Castration-resistant | 405959272 | 2688472 | 0.13 | 1025.68 |
| CRPC3 | Castration-resistant | 249794619 | 1652199 | 0.08 | 434.93 |
| CRPC4 | Castration-resistant | 688372873 | 4606813 | 0.22 | 125.14 |
| CRPC5 | Castration-resistant | 726262820 | 4943998 | 0.23 | 471.28 |
| T2 | Primary tumor | 216795596 | 1825954 | 0.07 | 18.44 |
| T3 | Primary tumor | 52607237 | 430824 | 0.02 | 29.80 |
| T4 | Primary tumor | 90964303 | 851256 | 0.03 | 35.09 |
| T5 | Primary tumor | 121933255 | 1065799 | 0.04 | 15.34 |
| T6 | Primary tumor | 178510313 | 1388939 | 0.06 | 32.76 |
| T7 | Primary tumor | 157194961 | 1307498 | 0.05 | 18.07 |
| CSPC1 | Castration-sensitive | 708455995 | 4830559 | 0.23 | 228.68 |
| CSPC1_2 | Castration-sensitive | 515057977 | 3484587 | 0.17 | 159.07 |
| CSPC1_3 | Castration-sensitive | 358658485 | 2424127 | 0.12 | 170.53 |
| CSPC2 | Castration-sensitive | 700160784 | 4722938 | 0.23 | 1155.77 |
| CSPC3 | Castration-sensitive | 311526284 | 2100676 | 0.10 | 822.12 |
| CSPC4 | Castration-sensitive | 156475411 | 1036261 | 0.05 | 327.65 |
| HT29_100 | Cell line | 378438431 | 2556458 | 0.12 | 429.74 |
| HT29_50 | Cell line | 492534283 | 3315189 | 0.16 | 160.56 |
| HT29_25 | Cell line | 500046817 | 3365938 | 0.16 | 51.59 |
| HT29_20 | Cell line | 379635341 | 2553600 | 0.12 | 33.26 |
| HT29_15 | Cell line | 256489327 | 1728922 | 0.08 | 25.07 |
| HT29_10 | Cell line | 346481033 | 2330290 | 0.11 | 13.85 |
| HT29_5 | Cell line | 363254426 | 2447148 | 0.12 | 8.50 |
| HT29_1 | Cell line | 171590517 | 1149503 | 0.06 | 7.75 |
| HT29_0 | Cell line | 189584022 | 1269274 | 0.06 | 2.72 |

^1^ Total bases sequenced; ^2^ Total Number of reads; ^3^ Mean coverage over whole genome; ^4^ genome-wide z-score

**Table S3**

**Genes and breakpoints included in the GB-panel**

| List of genes enriched (total coding sequence)^1^ | | | | |
| --- | --- | --- | --- | --- |
| Symbol | **Accession number: name** | |  | |
| *AKAP9* | NM_005751.4: A kinase (PRKA) anchor protein (yotiao) 9 | | | |
| *AKT1* | NM_005163.2: v-akt murine thymoma viral oncogene homolog 1 | | | |
| *APC* | NM_000038.5:Adenomatous polyposis of the colon gene | | | |
| *ARID1A* | NM_006015.4: AT rich interactive domain 1A (SWI-like) | | | |
| *ASXL1* | NM_015338.5: Additional sex combs like 1 (Drosophila) | | | |
| *ATM* | NM_000051.3: Ataxia telangiectasia mutated | | | |
| *AXIN1* | NM_003502.3: Axin 1 | | | |
| *BAX* | NM_004324.3, NM_138761.3: BCL2-associated X protein | | | |
| *BRAF* | NM_004333.4: v-raf murine sarcoma viral oncogene homolog B1 | | | |
| *BRCA1* | NM_007297.3: Familial breast/ovarian cancer gene 1 | | | |
| *BRCA2* | NM_000059.3: Familial breast/ovarian cancer gene 2 | | | |
| *BRIP1* | NM_032043.2: BRCA1 interacting protein C-terminal helicase 1 | | | |
| *CDH1* | NM_004360.3: Cadherin 1, type 1, E-cadherin (epithelial) (ECAD) | | | |
| *CDKN2A* | NM_058195.3: Cyclin-dependent kinase inhibitor 2A | | | |
| *CDKN2B* | NM_004936.3, NM_078487.2: Cyclin-dependent kinase inhibitor 2B (p15) | | | |
| *CHEK2* | NM_001005735.1: CHK2 checkpoint homolog (S. pombe) | | | |
| *CTNNA1* | NM_001903.2: Catenin (cadherin-associated protein), alpha 1, 102kDa | | | |
| *CTNNB1* | NM_001904.3: Catenin (cadherin-associated protein), beta 1 | | | |
| *DAPK3* | NM_001348.1: Death-associated protein kinase 3 | | | |
| *EGFR* | NM_201282.1, NM_005228.3: Epidermal growth factor receptor | | | |
| *EP300* | NM_001429.3: 300 kd E1A-Binding protein gene | | | |
| *ERBB2* | NM_004448.2: v-erb-b2 erythroblastic leukemia viral oncogene homolog 2 | | | |
| *FBXW7* | NM_001013415.1, NM_018315, NM_033632: F-box and WD-40 domain protein 7 | | | |
| *FGFR3* | NM_000142.4, NM_001163213.1: Fibroblast growth factor receptor 3 | | | |
| *GATA3* | NM_001002295.1: GATA binding protein 3 | | | |
| *GATA4* | NM_002052.3: GATA binding protein 4 | | | |
| *HNF1A* | NM_000545.5: HNF1 homeobox A | | | |
| *HRAS* | NM_001130442.1, NM_176795.3: Harvey rat sarcoma viral oncogene homolog | | | |
| *FOCAD* | NM_017794.3: Focadhesin (KIAA1797) | | | |
| *KLF6* | NM_001300.5: C Homo sapiens Kruppel-like factor 6 | | | |
| *KRAS* | NM_033360.2, NM_004985.3: Kirsten rat sarcoma 2 viral oncogene homolog | | | |
| *MAP2K4* | NM_003010.2: Mitogen-activated protein kinase kinase 4 | | | |
| *MDM2* | NM_002392.4: Mdm2 p53 binding protein homolog | | | |
| *MLH1* | NM_000249.3: E.coli MutL homolog gene | | | |
| *MLL3* | NM_170606.2: Myeloid/lymphoid or mixed-lineage leukemia 3 | | | |
| *MSH2* | NM_001258281.1, NM_000251.2: MutS homolog 2 (E. coli) | | | |
| *MSH6* | NM_000179.2: MutS homolog 6 (E. coli) | | | |
| *MUTYH* | NM_012222.2: MutY homolog (E. coli) | | | |
| *NF1* | NM_001042492.2: Neurofibromin 1 | | | |
| *NOTCH1* | NM_017617.3: Notch 1 | | | |
| *NRAS* | NM_002524.4: Neuroblastoma RAS viral (v-ras) oncogene homolog | | | |
| *PALB2* | NM_024675.3: Partner and localizer of BRCA2 | | | |
| *PDE4D* | NM_001165899.1, NM_001104631.1: Phosphodiesterase 4D, cAMP-specific | | | |
| *PIK3CA* | NM_006218.2: Phosphoinositide-3-kinase, catalytic, alpha polypeptide | | | |
| *PIK3R1* | NM_181523.2, NM_181524.1, NM_181504.3: Phosphoinositide-3-kinase, subunit 1a | | | |
| *PMS1* | NM_000534.4: PMS1 postmeiotic segregation increased 1 (S. cerevisiae) | | | |
| *PMS2* | NM_000535.5: PMS2 postmeiotic segregation increased 2 (S. cerevisiae) | | | |
| *PTEN* | NM_000314.4: Phosphatase and tensin homolog gene | | | |
| *RAD51C* | NM_058216.1: RAD51 homolog C (S. cerevisiae) | | | |
| *RB1* | NM_000321.2: Retinoblastoma gene | | | |
| *SMAD4* | NM_005359.5: Homolog of Drosophila Mothers Against Decapentaplegic 4 gene | | | |
| *SMO* | NM_005631.4: Smoothened, frizzled family receptor | | | |
| *SPOP* | NM_001007226.1: Speckle-type POZ protein | | | |
| *STK11* | NM_000455.4: Serine/threonine kinase 11 gene (LKB1) | | | |
| *TMEM135* | NM_022918.3: Transmembrane protein 135 | | | |
| *TP53* | NM_000546.5, NM_001126113.2: Tumor protein p53 | | | |
| Enrichment for fusion breakpoints | | | | **Chromosomal regions enriched^2^** |
| *ALK Intron 19* | | Anaplastic lymphoma kinase (Ki-1) | | Chr2:29446378_29448328 |
| *BCR Intron 8* | | Breakpoint cluster region | | Chr22:23615951_23626201 |
| *BCR Intron 13* | | Breakpoint cluster region | | Chr22:23631801_23632551 |
| *BCR Intron 14* | | Breakpoint cluster region | | Chr22:23632601_23634751 |
| *BRAF Intron7-10* | | v-raf murine sarcoma viral oncogene homolog B1 | | chr7:140481465_140500165 |
| *EGFR Intron 7* | | Epidermal growth factor receptor | | chr7:55221824_55223574 |
| *ETV1 Intron 3-4* | | Ets variant gene 1 | | chr7:14026293_14028643 |
| *ETV4 Intron 8* | | Ets variant gene 4 | | chr17:41607506_41610056 |
| *ETV5 Intron 6-7* | | Ets variant gene 5 | | chr3:185783852_185798852 |
| *ETV6 Intron 5-6* | | Ets variant gene 6 | | chr12:12022887_12038887 |
| *EWSR1 Intron 8-13* | | Ewing sarcoma breakpoint region 1 | | chr22:29684747_29694747 |
| *MLL Intron 6-9* | | Myeloid/lymphoid or mixed-lineage leukemia | | chr11:118350904_118355604 |
| *RAF1 Intron 5-9* | | v-raf-1 murine leukemia viral oncogene homolog 1 | | chr3:12641301_12650301 |
| *RARA Intron 2* | | Retinoic acid receptor, alpha | | chr17:38487622_38504572 |
| *RET Intron 9-11* | | Ret proto-oncogene | | chr10:43608366_43612066 |
| *TMPRSS2 Intron* | | Transcription factor 7-like 2 | | chr21:42866486_42880036 |
| *VTI1A Intron* | | Transmembrane protease, serine 2 | | chr10:114224405_114298055 |
| *TCF7L2 Intron* | | Transcription factor 7-like 2 | | chr10:114799858_114903708 |

^1^ RefSeq accession numbers that were used for Custom SureSelect design to cover all coding exons of different transcript variants

^2^ Hg19 coordinates that were used for Custom SureSelect design to cover common breakpoint regions in prostate cancer

**Table S4**

**Summary of next-generation plasma sequencing employing the GB-panel and deep-sequencing.**

|  | Targeted enrichment of GB-Panel | | | | | | | Deep sequencing | |
| --- | --- | --- | --- | --- | --- | --- | --- | --- | --- |
| Sample | **Total bases^1^** | **Total reads^2^** | **Coverage^3^** | **% >50x^4^** | **Mutation^5^** | **Split reads^6^** | ***TMPRSS-ERG^7^*** | **Coverage^8^** | **% mutated^9^** |
| CRPC1 | 589497941 | 3946440 | 93.1 | 68.2 | none | 3 | yes | NA | NA |
| CRPC2 | 541974424 | 3621506 | 100.9 | 68.6 | MLL3 p.R4763Q | 0 | no | 356289 | 50.5 |
| CRPC3 | 975339510 | 6553664 | 232.7 | 75.1 | none | 0 | no | NA | NA |
| CRPC5 | 1691467638 | 11419958 | 314.7 | 72.3 | TP53 p.R116Q | 1 | yes | 442294 | 54.5 |
| CSPC1 | 1507782701 | 10148900 | 293.0 | 73.5 | none | 8 | yes | NA | NA |
| CSPC2 | 2227382648 | 14956676 | 470.3 | 77.6 | BRCA2 p.A2525fs HNF1A p.P424A | 0 | no | 354477 485467 | 56.0 39.5 |
| CSPC4 | 569577168 | 3816700 | 86.5 | 67.3 | BRCA1 p.Q928R | 1 | yes | 307592 | 30.7 |

^1^ Total bases sequenced; ^2^ Total Number of reads;^3^ Mean Coverage over whole target region; ^4^ percentage of targeted bases covered more than 50x; ^5^ Identified mutation; none, no mutation identified; ^6^ Number of split read fragments covering *TMPRRS-ERG* fusion; ^7^ Presence of *TMPRRS-ERG* fusion;^8^ Fold coverage of deep sequencing of fragments harboring the mutation identified with the GB-Panel;^9^ Percentage of mutated reads indicating the amount of tumor-specific DNA in plasma; NA, not available
