# Supplementary figures and images for: Tumor-associated copy number changes in the circulation of patients with prostate cancer identified through whole-genome sequencing
Source: Genome Med. 2013 Apr 5;5(4):30. doi: 10.1186/gm434 (PMC3707016; doi:10.1186/gm434)

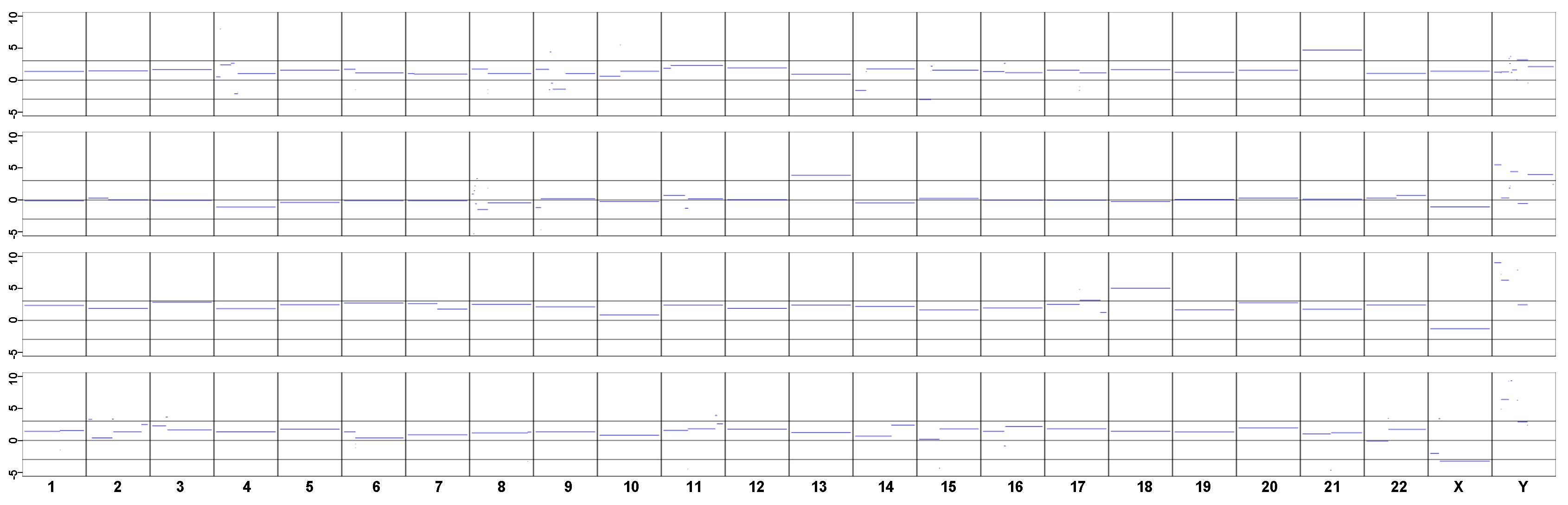

Supplement: Additional file 2 — Plasma DNA analyses from pregnant women. Plasma DNA analyses from maternal blood with pregnancies with a trisomy 21 fetus (first panel), a trisomy 13 fetus (second panel), a trisomy 18 fetus (third panel), and an euploid fetus (fourth panel) (X-axis: Chromosome; Y-axis: z-score). [file gm434-S2.TIFF]

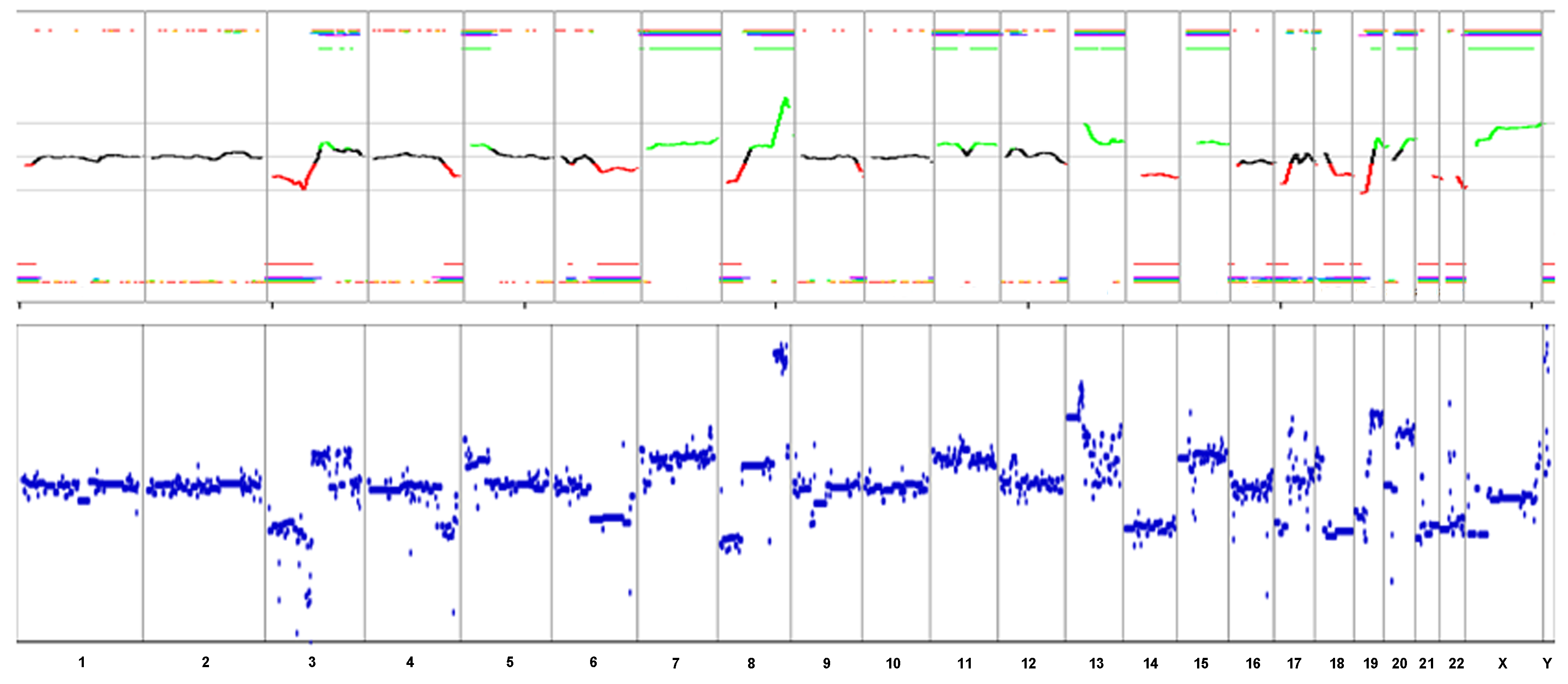

Supplement: Additional file 3 — Copy-number status of the HT29 cell line. The upper panel illustrates the array-CGH profile, the lower panel the profile obtained with our next-generation sequencing approach. Both panels illustrate the copy number profile with undiluted, that is, 100%, DNA. In the array-CGH profile the multicolor bar codes at the top or bottom of the ratio profiles illustrate the results obtained during the iterative calculations with various window sizes, the single green and red bars summarize the regions which were gained or lost based on all calculations (for details see [44]). Black parts in the profile represent balanced regions, lost regions appear in red, and gained regions in green. [file gm434-S3.TIFF]

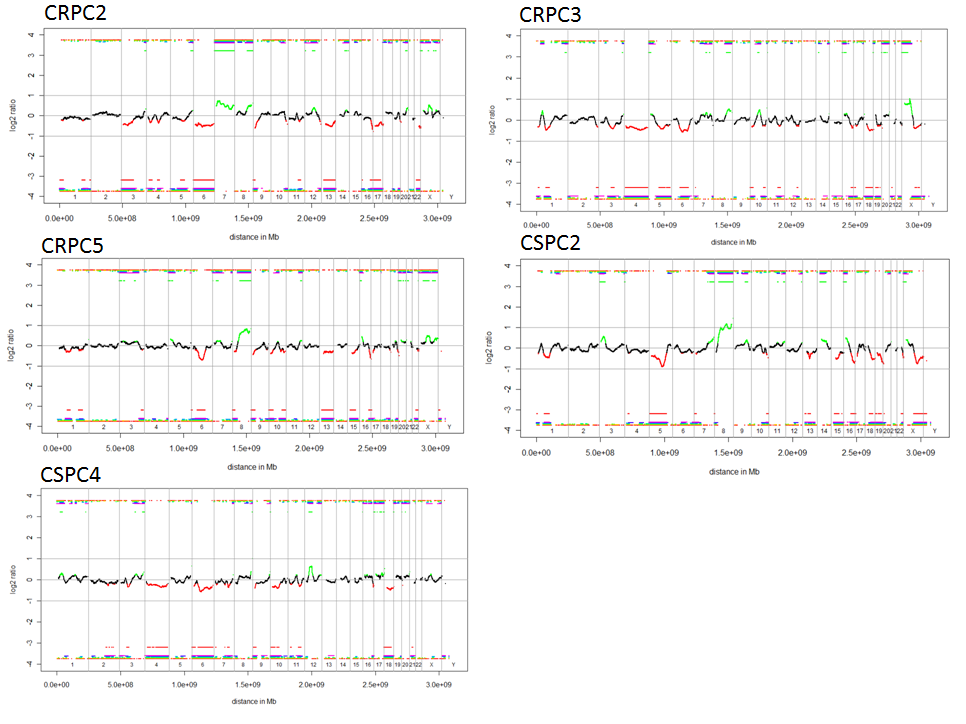

Supplement: Additional file 4 — Array-CGH evaluations as control for our plasma-Seq approach: Array-CGH profiles of plasma samples CRPC2, CRPC3, CRPC5, CSPC2, and CSPC4. For all array-CGH profiles the multicolor bar codes at the top or bottom of the ratio profiles illustrate the results obtained during the iterative calculations with various window sizes, the single green and red bars summarize the regions which were gained or lost based on all calculations (for details see [44]). Black parts in the profile represent balanced regions, lost regions appear in red, and gained regions in green. Previously we had already demonstrated the use of array-CGH analyses for the analysis of plasma DNA [33]. The array-CGH profiles show a great concordance with those obtained with plasma-Seq. [file gm434-S4.TIFF]

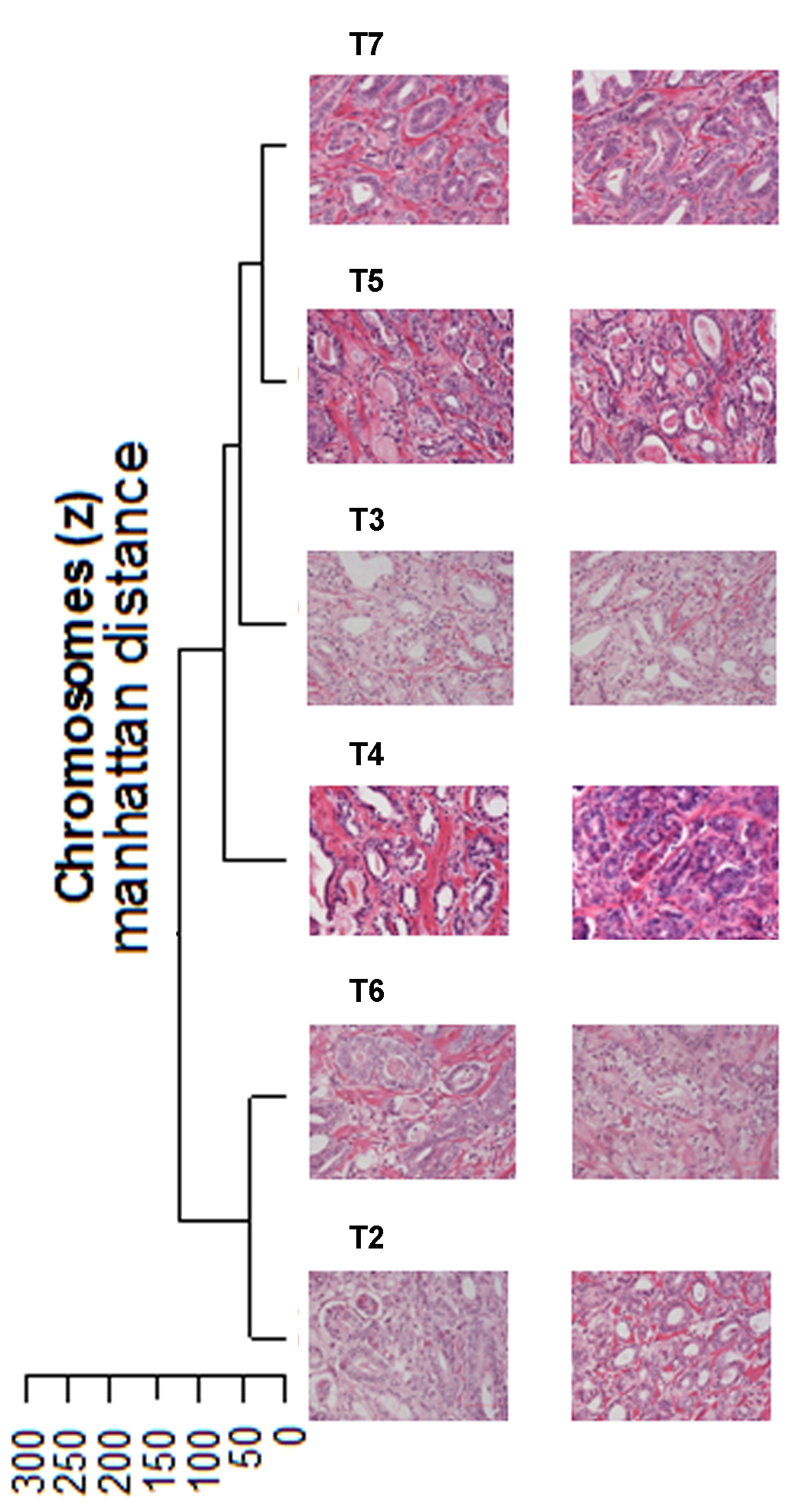

Supplement: Additional file 5 — Histology samples from the primary tumor of patient CRPC1. The six different samples are arranged according to the hierarchical clustering (Manhattan distances of chromosomal z-scores) from Figure 5, the corresponding part of the tree is shown to the left. From each sector the most common (left) and second most common (right) patterns are shown. Relating the hierarchical clustering of the chromosomal alterations in the various sectors of the primary tumor to morphological features, no clear picture emerges. Regarding the growth pattern, T2 and T3 seem closely related as are T4 and T5, which is not reflected in the clustering analysis of the chromosomal alterations. Based on nuclear staining features, T7 seems similar to T2 and T3. T6 also shares features of T2 and T3. Taking into account the changes detected in circulating DNA, the most likely explanation is a complex multifocal disease resulting in a complex morphological as well as genetic pattern. [file gm434-S5.TIFF]

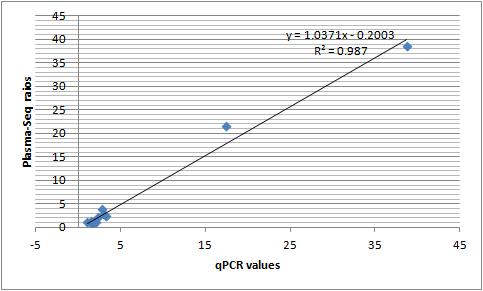

Supplement: Additional file 6 — Validation of the AR copy number status with qPCR for plasma samples CRPC1, CRPC2, CRPC5, CSPC1, CSPC1_2, and CSPC2 showing a very close correlation between the plasma-Seq and the qPCR values. [file gm434-S6.TIFF]
